# Nucleic Acid Therapeutics in Rare Disease: From Precision Medicine to Programmable Platforms

**DOI:** 10.1002/jimd.70262

**Published:** 2026-11-02

**Authors:** Tomasz Z. Tomkiewicz, Siyamini Sivananthan, Paul Gissen, Haiyan Zhou

**Affiliations:** ^1^ Genetics and Genomic Medicine Research and Teaching Department Great Ormond Street Institute of Child Health, University College London London UK; ^2^ NIHR Great Ormond Street Hospital Biomedical Research Centre London UK

**Keywords:** antisense oligonucleotides, gene editing, individualised therapy, mRNA therapeutics, nucleic acid therapeutics, precision medicine, rare disease, RNA interference

## Abstract

Nucleic acid therapeutics (NATs), including antisense oligonucleotides (ASOs), small interfering RNAs (siRNAs), messenger RNA (mRNA) therapeutics and programmable editing technologies, are reshaping the therapeutic landscape for inherited metabolic and genetic disease. Unlike conventional small molecules or biologics, NATs enable sequence‐directed modulation of target gene expression and therefore provide unprecedented flexibility for targeting pathogenic mechanisms previously considered undruggable. Recent clinical successes have established proof of concept for multiple NAT modalities across neurological, hepatic, muscular and metabolic disorders. However, broader implementation remains constrained by challenges in tissue‐specific delivery, biomarker validation, natural‐history infrastructure and evidence generation in ultra‐rare populations. In parallel, increasing platform reproducibility is beginning to challenge traditional product‐based regulatory paradigms. This review examines the principal classes of NATs and proposes a practical framework for modality selection based on disease biology, tissue accessibility, durability, reversibility and clinical context. We discuss key translational bottlenecks beyond delivery, including biomarker–outcome relationships, small‐population trial design and the limitations of conventional evidentiary frameworks in rare disease. A central theme of this review is the growing transition from isolated precision medicines towards increasingly programmable therapeutic platforms. We explore how advances in extrahepatic delivery, computational oligonucleotide design, artificial intelligence‐assisted optimisation and programmable editing technologies may progressively support more scalable and individualised therapeutic development. Finally, we discuss emerging regulatory frameworks that may better accommodate platform‐level predictability while maintaining rigorous evaluation of sequence‐specific and disease‐specific risks.

## 1 Introduction

Nucleic acid therapeutics (NATs) have emerged as one of the most important developments in modern precision medicine. NATs directly target RNA or DNA through programmable Watson–Crick base pairing, enabling sequence‐specific modulation of transcript processing, RNA stability, protein restoration or genomic sequence correction [1]. Major modalities include antisense oligonucleotides (ASOs), small interfering RNAs (siRNAs), messenger RNA (mRNA) therapeutics and RNA‐editing systems [2, 3], expanding therapeutic options beyond conventional pharmacology.

Rare genetic diseases are well suited for NAT development because monogenic disorders often link genotype directly to phenotype. Landmark approvals such as *nusinersen* for spinal muscular atrophy (SMA) [4], *patisiran* and *vutrisiran* for hereditary transthyretin amyloidosis (hATTR) [5, 6] and *lumasiran* for primary hyperoxaluria Type 1 [7] have established clinical proof of concept for multiple NAT modalities (Figure 1). These successes also expose scientific, translational and regulatory challenges, especially in ultra‐rare diseases where patient numbers and evidentiary expectations are often misaligned [8, 9, 10].

**FIGURE 1:**
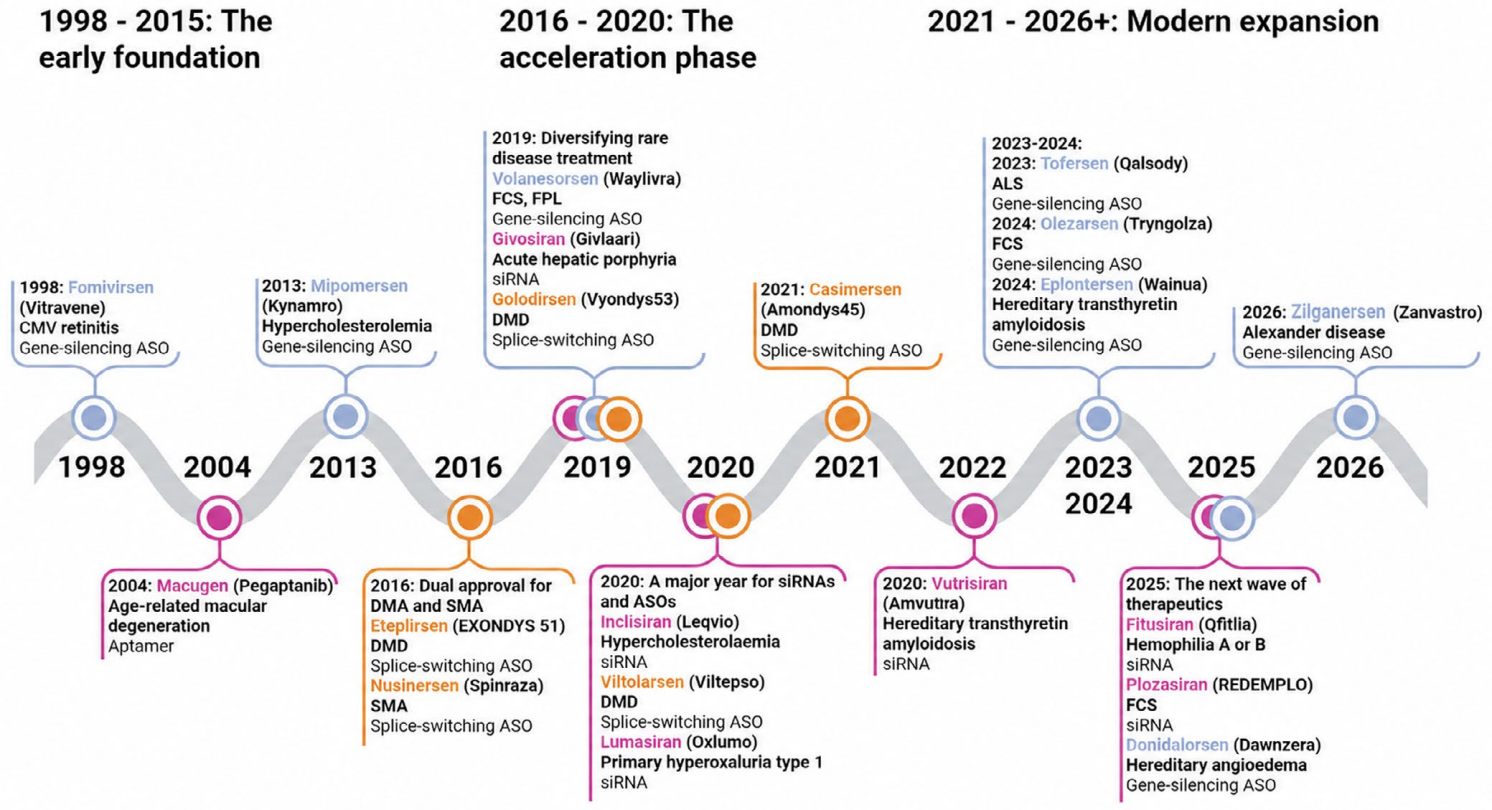
Timeline of market‐approved oligonucleotide therapeutics. Colours indicate modality class: Splice‐switching ASOs (yellow), gene‐silencing ASOs (blue) and siRNAs (purple). ALS, amyotrophic lateral sclerosis; DMD, Duchenne muscular dystrophy; FCS, familial chylomicronaemia syndrome; FPL, familial partial lipodystrophy; SMA, spinal muscular atrophy.

NATs modalities differ in persistence, reversibility, delivery, re‐dosing feasibility and long‐term risk. Rather than existing as isolated technologies, NAT increasingly form a continuum of programmable RNA modulation extending from transient transcript suppression to transcript correction and restoration.

This review examines the current state of major NAT modalities and proposes a framework for therapeutic selection in rare‐diseases based on disease biology, tissue accessibility, durability, reversibility and patient context. It also discusses translational bottlenecks, regulatory challenges, emerging technologies and future directions that may shape the next phase of rare‐disease precision medicine.

## 2 Classes of Nucleic Acid Therapeutics

### 2.1 Antisense Oligonucleotides Therapeutics

ASOs are short, single‐stranded synthetic oligonucleotides, usually 15 to 30 nucleotides in length, that bind complementarily to pre‐mRNA or mRNA through Watson–Crick base pairing [11]. Their therapeutic activity depends heavily on backbone chemistry and sugar modification, which influence nuclease resistance, protein binding, tissue biodistribution, cellular uptake and toxicity.

Modern therapeutic ASOs commonly incorporate phosphorothioate backbones and 2′‐sugar modifications such as 2′‐*O*‐methyl (2′‐OMe) and 2′‐*O*‐methoxyethyl (MOE). More extensively modified chemistries, including locked nucleic acids (LNA), constrained ethyl (cEt) residues and phosphorodiamidate morpholino oligomers (PMOs), further improve stability and pharmacokinetic profile [11, 12]. Therapeutic ASOs operate through two principal mechanisms of action [13, 14]. Gapmer ASOs are used for transcript degradation by recruiting endogenous RNase H1 to target pre‐ and mRNA, whereas steric‐blocking ASOs alter pre‐mRNA splicing or block translation for target genes modulation without RNA cleavage [11, 12].

Clinical experience has demonstrated the mechanistic versatility of ASOs across multiple diseases. *Nusinersen* promotes inclusion of *SMN2* exon 7 and restore full‐length SMN protein expression in SMA [4], whereas *tofersen* reduces mutant *SOD1* transcripts in amyotrophic lateral sclerosis (ALS) through RNase H‐mediated mRNA degradation [15]. Exon‐skipping ASOs such as *eteplirsen*, *golodirsen*, *viltolarsen* and *casimersen* restore the dystrophin reading frame in Duchenne muscular dystrophy (DMD) [16]. These examples present both the flexibility and limitations of ASO platforms. Although chemistry and manufacturing principles can often be generalised across programmes, pharmacology remains strongly influenced by mechanism of action, sequence, target biology, tissue accessibility and route of administration. The clinical maturity of these modalities varies across target organs, with the strongest approval history currently seen in liver‐directed and locally delivered oligonucleotide therapies (Table 1).

**TABLE 1:** Approved oligonucleotide therapeutics organised by primary target organ.

Drug (brand)	Year of approval	Mode of action	Target/indication	Key target organ
Mipomersen (Kynamro)	2013	Gene‐silencing ASO—blocks ApoB‐100 mRNA translation via RNase H	ApoB‐100 Homozygous familial hypercholesterolaemia	Liver
Givosiran (Givlaari)	2019	siRNA (GalNAc)—RISC‐mediated ALAS1 mRNA degradation	ALAS1 Acute hepatic porphyria	Liver
Inclisiran (Leqvio)	2020	siRNA (GalNAc)—RISC‐mediated PCSK9 mRNA degradation	PCSK9 Hypercholesterolaemia/mixed dyslipidaemia	Liver
Lumasiran (Oxlumo)	2020	siRNA (GalNAc)—silences HAO1 mRNA in hepatocytes	HAO1 Primary hyperoxaluria Type 1	Liver
Vutrisiran (Amvuttra)	2022	siRNA (GalNAc)—silences hepatic TTR mRNA	TTR Hereditary transthyretin amyloidosis (hATTR)	Liver
Inotersen (Tegsedi)	2018	Gene‐silencing ASO—RNase H‐mediated TTR mRNA cleavage	TTR hATTR polyneuropathy	Liver
Volanesorsen (Waylivra)	2019	Gene‐silencing ASO—degrades ApoCIII mRNA via RNase H	ApoCIII FCS/familial partial lipodystrophy	Liver
Fitusiran (Alhemo)	2025	siRNA (GalNAc)—silences hepatic antithrombin (SERPINC1) mRNA	Antithrombin Haemophilia A & B	Liver
Olezarsen (Tryngolza)	2024	Gene‐silencing ASO (GalNAc)—degrades ApoCIII mRNA	ApoCIII Familial chylomicronaemia syndrome (FCS)	Liver
Eplontersen (Wainua)	2024	Gene‐silencing ASO (GalNAc)—RNase H‐mediated TTR mRNA degradation	TTR hATTR polyneuropathy	Liver
Nusinersen (Spinraza)	2016	Splice‐switching ASO—promotes SMN2 exon 7 inclusion; intrathecal delivery	SMN2 Spinal muscular atrophy (SMA)	CNS
Tofersen (Qalsody)	2023	Gene‐silencing ASO—RNase H‐mediated SOD1 mRNA degradation; intrathecal	SOD1 ALS (SOD1 mutation)	CNS
Zilganersen (Zanvastro)	2026	Gene‐silencing ASO—RNase H‐mediated GFAP mRNA degradation; intrathecal	GFAP · Alexander disease	CNS
Fomivirsen (Vitravene)	1998	Gene‐silencing ASO—blocks CMV IE2 mRNA translation; intravitreal	CMV IE2 CMV retinitis (HIV patients)	Retina
Pegaptanib (Macugen)	2004	RNA aptamer—binds and neutralises extracellular VEGF165; intravitreal	VEGF165 Neovascular age‐related macular degeneration	Retina
Eteplirsen (Exondys 51)	2016	Splice‐switching ASO—skips dystrophin exon 51, restores reading frame	DMD exon 51 Duchenne muscular dystrophy	Muscle
Golodirsen (Vyondys 53)	2019	Splice‐switching ASO—skips dystrophin exon 53, restores reading frame	DMD exon 53 Duchenne muscular dystrophy	Muscle
Viltolarsen (Viltepso)	2020	Splice‐switching ASO—skips dystrophin exon 53, restores reading frame	DMD exon 53 Duchenne muscular dystrophy	Muscle
Casimersen (Amondys 45)	2021	Splice‐switching ASO—skips dystrophin exon 45, restores reading frame	DMD exon 45 Duchenne muscular dystrophy	Muscle

### 2.2 RNA Interference Therapeutics

siRNAs are short double‐stranded RNA molecules, typically 19 to 23 nucleotides in length, that induce post‐transcriptional gene silencing through the RNA‐induced silencing complex (RISC) [17, 18]. Following cellular uptake, the guide strand is incorporated into RISC, directing Argonaute‐2 (AGO2) to complementary mRNA targets and inducing sequence‐specific cleavage. A single loaded complex can degrade multiple transcripts, amplifying silencing efficiency [18]. Chemical stabilisation through 2′‐OMe, 2′‐fluoro and phosphorothioate modifications has substantially improved siRNA pharmacokinetics and reduced innate immune activation [12, 19, 20]. The major translational breakthrough for siRNA therapeutics has been the development of N‐acetylgalactosamine (GalNAc) conjugation, which enables targeted hepatocyte delivery via the asialoglycoprotein receptor [21].

GalNAc‐conjugated siRNAs now represent the most mature example of scalable systemic NAT delivery. Multiple market‐approved NAT products, including *givosiran*, *lumasiran*, *vutrisiran* and *inclisiran*, demonstrate reproducible hepatic delivery, durable transcript suppression and infrequent dosing schedules [6, 7, 21, 22, 23]. Importantly, GalNAc‐siRNA therapeutics provide one of the clearest example of partial platform validation within NAT development. Shared chemistry, tissue targeting and pharmacokinetic profile support increasing regulatory confidence across related programmes. However, this success remains largely confined to hepatic indications and reproducible extrahepatic delivery remains substantially less mature [21].

### 2.3 Messenger RNA Therapeutics

mRNA therapeutics enables transient intracellular production of therapeutic proteins without genomic integration [24]. Following delivery into the cytoplasm, mRNA is translated by endogenous cellular machinery to produce a target protein [25, 26]. The development of nucleoside‐modified mRNA encapsulated within lipid nanoparticles (LNPs) demonstrated that mRNA can be manufactured and delivered at scale with acceptable short‐term safety profiles. This established mRNA as a clinically viable therapeutic platform [27, 28].

In rare disease, mRNA therapeutics are currently most advanced in hepatic disorders, including propionic acidaemia, methylmalonic acidaemia, argininosuccinic aciduria and ornithine transcarbamylase deficiency [29, 30, 31, 32]. These programmes exploit the relative maturity of liver‐directed LNP delivery. However, chronic protein replacement presents distinct translational challenges compared with vaccination. Repeated systemic dosing, cumulative innate immune activation, durability limitations, manufacturing burden and long‐term tolerability remain important barriers [33].

Emerging approaches, including self‐amplifying RNA, circular RNA and optimised cap structures, aim to extend expression duration and reduce dose frequency, although durable clinical benefit in rare disease has yet to be fully established [34, 35, 36, 37].

### 2.4 Gene and RNA Editing Therapeutics

Programmable editing technologies differ fundamentally from ASO, siRNA or mRNA therapeutics because they aim to directly modify pathogenic nucleic acid sequences rather than transiently modulate gene expression. These strategies include both genome editing, which alters DNA sequence and RNA editing, which enables targeted correction of RNA transcripts without permanent genomic modification [38, 39].

CRISPR‐Cas9 genome editing employs a guide RNA to direct site‐specific DNA cleavage, after which endogenous repair pathways generate either disruptive changes via non‐homologous end joining or precise edits through homology‐directed repair [38, 39]. More recent innovations, including base editing and prime editing, enable targeted nucleotide conversions without introducing double‐strand DNA breaks, thereby reducing the risk of unintended genomic damage [38, 40, 41]. Clinical translation has now been achieved in selected settings. *Exagamglogene autotemcel* showed that ex vivo CRISPR‐edited autologous cell therapy can deliver substantial clinical benefit in sickle cell disease [42], while early in vivo studies, such as *NTLA‐2001*, demonstrated the feasibility of liver‐directed genome editing in transthyretin amyloidosis [43].

In parallel, RNA editing technologies, particularly adenosine deaminases acting on RNA (ADARs)‐mediated systems, are emerging as potentially reversible alternatives to permanent genome editing [44]. These approaches enable site‐specific modification of RNA transcripts without altering genomic DNA and may therefore offer a more flexible safety profile in selected contexts, particularly for missense variants and paediatric disorders [45]. However, clinical translation remains early and major challenges persist, including delivery efficiency, editing precision, durability and off‐target effects.

## 3 A Modality Selection Framework for Rare Disease

Selecting an appropriate NAT modality for rare disease is not a simple ranking exercise among ASO, siRNA, mRNA and programmable editing. Each modality differs fundamentally in mechanism, reversibility, durability, delivery requirement and evidentiary expectations. Consequently, the optimal strategy depends on how well a modality aligns with disease biology, tissue accessibility, therapeutic window and long‐term treatment context rather than on overall technological maturity (Figure 2).

**FIGURE 2:**
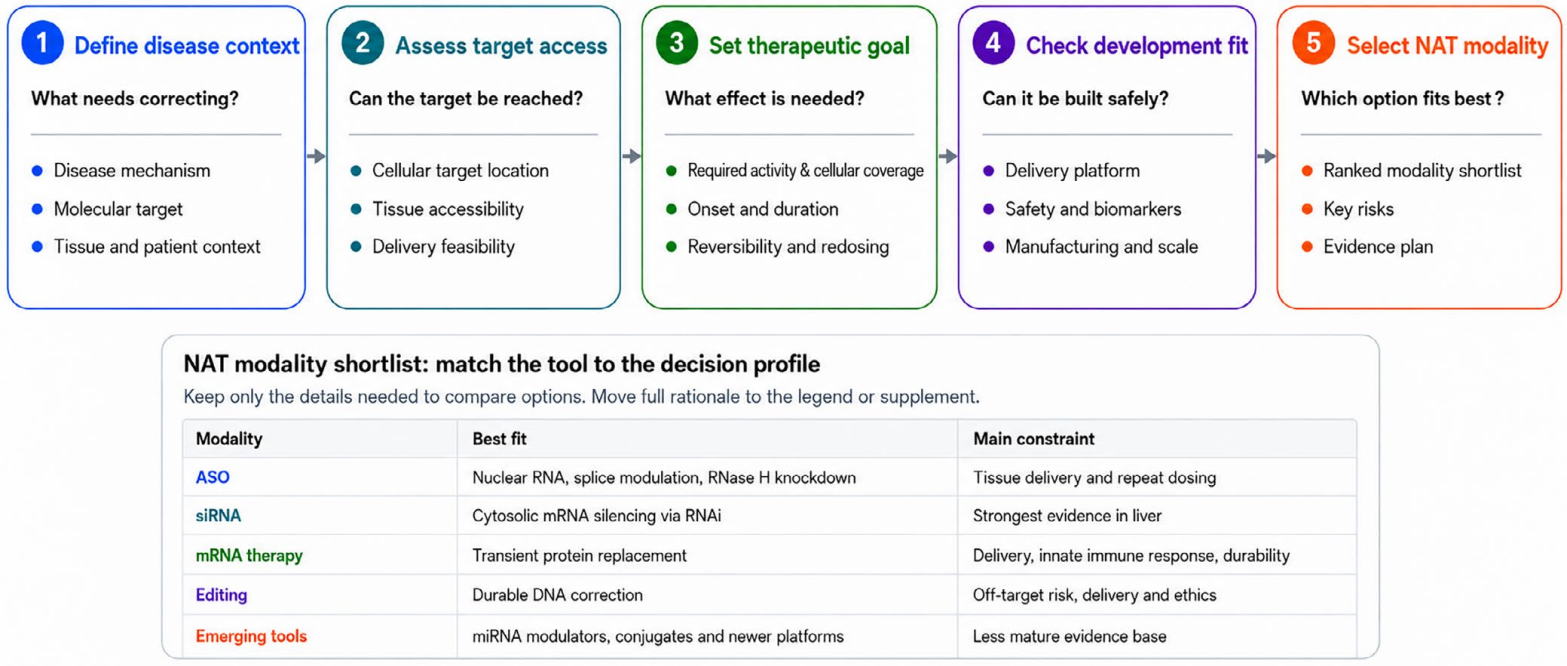
Framework for nucleic acid therapeutic (NAT) modality selection in rare disease. The framework uses five decision steps to define disease biology, identify an appropriate therapeutic target tissue, establish the functional activity and cellular coverage required for benefit, assess development feasibility and select the best‐fit NAT modality.

Modality selection should address four associated questions: does the mechanism match the disease biology; what functional activity and cellular coverage are required; can the target tissue be reached efficiently; and does the therapy fit the clinical context? These factors also influence the extent to which regulators can rely on prior platform knowledge, an issue discussed further in Sections 5 and 6.

### 3.1 Match Therapeutic Mechanism With Disease Biology

The first consideration in modality selection is whether the intervention appropriately addresses the underlying disease molecular mechanism. Variants that disrupt splicing, generate toxic RNA species or produce dominant gain‐of‐function effects are often well suited to transcript‐targeting approaches such as ASOs or siRNAs [14, 46]. In contrast, disorders caused by loss‐of‐function may require restoration strategies such as mRNA replacement or genomic correction.

This mechanistic distinction is particularly important in rare genetic disease, where the relationship between genotype and phenotype is often well defined. Splice‐switching ASOs can restore productive transcript processing in diseases such as SMA and DMD, whereas siRNA‐mediated knockdown is more appropriate when reduction of a toxic transcript is required. By contrast, mRNA therapeutics aim to transiently restore missing protein expression without altering genomic DNA and may therefore be particularly attractive for inherited metabolic disorders requiring repeated protein replacement.

Genome editing may be appropriate when durable correction offers a clear clinical advantage over reversible alternatives and when the expected benefit justifies the risks associated with irreversible genomic modifications, taking into account disease mechanism, available treatments, tissue accessibility, age, disease stage and long‐term safety [41, 47]. In an infant with severe carbamoyl phosphate synthetase 1 (CPS1) deficiency, personalised lipid nanoparticle‐delivered adenine base‐editing showed that highly individualised editing can be feasible in selected ultra‐rare metabolic disorders [48].

The magnitude and distribution of molecular correction required should also be considered. For some enzyme deficiencies, restoration of only a small proportion of normal protein activity may be sufficient to restore metabolic flux, whereas other disorders require correction across a much larger fraction of affected cells [49, 50]. This depends partly on whether pathogenic metabolites can move between cells: diffusible metabolites may allow corrected cells to compensate for neighbouring uncorrected cells, whereas predominantly cell‐autonomous defects require broader cellular coverage. These contrasting principles are well illustrated in CPS1 deficiency and primary hyperoxaluria Type 1 [48, 51].

### 3.2 Align Therapeutic Strategy With Clinical Context

Clinical fit also depends on disease stage and therapeutic window. In SMA, outcomes are better when treatment begins before or soon after symptom onset, illustrating how therapeutic window and disease progression shape efficacy [52]. Five‐year NURTURE follow‐up showed sustained survival and motor improvement following presymptomatic *nusinersen* treatment, reinforcing the importance of early intervention in progressive neurogenetic disease [53, 54].

Residual tissue function and disease stage are equally important. Leber congenital amaurosis Type 10 (LCA10), caused by the *CEP290* c.2991 + 1655A > G deep intronic variant, represents a strong mechanistic match for splice‐switching therapy because the pathogenic mechanism is transcript mediated and the eye is accessible to local treatment. However, follow‐up analyses of sepofarsen demonstrated that molecular correction did not uniformly translate into higher‐order visual improvement, highlighting the importance of residual retinal integrity and timing of intervention [55]. Therapeutic success therefore depends not only on correcting the molecular defect but also on whether sufficient functional tissue remains to generate meaningful clinical benefit.

Practical treatment burden also matters. In hATTR polyneuropathy, *patisiran* requires intravenous infusion every 3 weeks [5], whereas *vutrisiran* is given subcutaneously every 3 months [6]. NICE estimated about 17–18 annual visits for *patisiran* versus four for *vutrisiran* [56]. Such differences affect adherence, caregiver burden, healthcare resources and quality of life in chronic rare disease.

### 3.3 Efficient and Sustainable Tissue Targeting

Once the required therapeutic activity and cellular coverage have been defined, the next question is whether these requirements can be achieved through safe and sustainable tissue delivery. Delivery remains one of the principal translational constraints for NATs [57]. Hepatic targeting currently represents the most mature example of successful systemic NAT delivery. GalNAc‐siRNA conjugates enable efficient hepatocyte uptake following subcutaneous administration and now underpin several approved liver‐directed therapies. *Inclisiran*, for example, reduces LDL‐cholesterol by approximately 50% with only twice‐yearly maintenance dosing [58, 59], demonstrating how optimised delivery chemistry can confer both durable pharmacological effect and reduced treatment burden [58, 59]. Similar principles are evident in hereditary transthyretin amyloidosis (hATTR), where the GalNAc‐conjugated ASO *eplontersen* allows once‐monthly subcutaneous administration compared with weekly dosing for *inotersen* [58, 59].

Importantly, the therapeutic target does not always need to coincide with the organ showing the dominant clinical pathology. In a mouse model of gyrate atrophy, liver‐directed restoration of ornithine aminotransferase reduced circulating and ocular ornithine and improved retinal pathology [60]. Although this study used AAV‐mediated gene transfer, it demonstrates the broader principle that correcting metabolism in an accessible organ may protect extrahepatic tissues by reducing systemic exposure to a pathogenic metabolite.

Beyond the liver, however, tissue access remains substantially more challenging [61]. Central nervous system (CNS) delivery is clinically feasible by repeated intrathecal (IT) administration, which is invasive and resource intensive. In one paediatric SMA series, 104 lumbar punctures were performed in 26 patients over 9 months, illustrating both the feasibility and burden of care [62]. Similar challenges remain for skeletal muscle, lung and other extrahepatic tissues where systemic uptake is limited and repeated local administration may not be practical. Consequently, tissue accessibility remains one of the major determinants of which NAT modalities are currently scalable in clinical practice.

## 4 Translational Bottlenecks Beyond Delivery

Beyond delivery, NAT translation may be constrained by weak natural‐history data, uncertain biomarker‐clinical outcome relationship and the difficulties of generating robust evidence in very small populations. These linked problems require more adaptable evidentiary frameworks.

### 4.1 Natural History as Evidentiary Infrastructure

Rare‐disease NAT development frequently depends on estimating what would have happened in the absence of treatment. Because conventional randomised controlled trials are often impractical in ultra‐rare populations, many programmes rely on single‐arm studies supported by external controls derived from registries, natural‐history cohorts or retrospective case series [63, 64]. The reliability of these approaches depends heavily on how well untreated disease progression has been characterised.

Natural‐history infrastructure is often incomplete because longitudinal datasets take years to build, while therapeutic development can advance rapidly once a tractable molecular target is identified. Trials may therefore begin before prospective natural‐history cohorts mature, forcing reliance on fragmented retrospective datasets collected with inconsistent criteria, follow‐up and outcomes.

Poorly matched historical controls make treatment effects harder to separate from baseline heterogeneity and variable progression. DMD exon‐skipping programmes therefore relied heavily on dystrophin restoration because functional trajectories vary across patients [65]. By contrast, the APOLLO trial used a randomised placebo‐controlled design for *patisiran* in hATTR, preserving contemporaneous comparison where progression and outcome are less predictable [5].

Natural‐history limitations are often greatest in the very diseases where programmable therapeutics are most attractive: ultra‐rare, genetically heterogeneous and rapidly progressive disorders. Natural‐history studies should therefore be viewed not as supportive background information, but as essential evidentiary infrastructure for rare‐disease therapeutic development.

### 4.2 Biomarkers Between Target Engagement and Clinical Benefit

NAT development often benefits from mechanistically direct pharmacodynamic readouts, including transcript reduction, splice correction, protein restoration or metabolite normalisation. However, demonstrating molecular target engagement does not necessarily establish meaningful clinical benefit.

In many programmes, direct assessment of target engagement within affected tissues is difficult or impractical. Biomarker strategies therefore frequently rely on downstream surrogate measures rather than direct confirmation of therapeutic activity at the disease site. In primary hyperoxaluria Type 1, for example, *lumasiran* development relied on urinary and plasma oxalate reduction as accessible downstream indicators of hepatic target engagement [7], although these measures only partially reflect ongoing oxalate synthesis, as discussed below. In DMD, dystrophin restoration can be measured directly through muscle biopsy, but repeated sampling is invasive and difficult to standardise at scale [65].

The evidentiary challenge lies not only in whether a biomarker can be measured, but also in what it can reliably predict. Pharmacodynamic biomarkers may demonstrate biological activity, whereas surrogate endpoints aim to predict clinical benefit. Clinical outcomes, by contrast, assess how patients feel, function or survive [66, 67]. In rare‐disease NAT development, molecular effects are often established before robust relationships between biomarker change and long‐term patient benefit have been fully defined.

This uncertainty is particularly apparent in Huntington's disease. Lowering mutant huntingtin in CSF supports biological target engagement, yet its relationship to meaningful clinical improvement remains uncertain [68]. Primary hyperoxaluria Type 1 also illustrates limitations in biomarker interpretation. Urinary and plasma oxalate do not directly isolate ongoing endogenous oxalate production because measured concentrations are influenced by renal clearance and exchange with tissue stores. Stable‐isotope tracing can provide a more direct assessment of pathway flux and distinguish ongoing production from changes in metabolite concentration [51].

The challenge is compounded by the absence of validated clinical outcome measures in many rare diseases. Functional assessments and patient‐reported outcomes are frequently adapted from related disorders rather than specifically developed for the disease under study [69]. Weak clinical endpoints therefore also make biomarker validation more difficult, creating a reciprocal evidentiary problem.

### 4.3 Adapting Trial Design to Small Populations

Rare‐disease NAT development has to generate credible evidence in populations too small for conventional clinical‐trial paradigms. Alternative frameworks in clinical trial design are increasingly used, but each depends heavily on disease biology, outcome behaviour and the expected magnitude of treatment effect (Table 2).

**TABLE 2:** Common clinical trial design frameworks in rare disease nucleic acid therapeutic development.

Design framework	Strengths in rare disease NATs	Key limitations	Best suited to
Single‐arm with historical or external control	Feasible in very small populations; avoids exposing scarce patients to a control arm	Sensitive to natural‐history bias and baseline heterogeneity	Large expected effects, relatively predictable progression, strong supportive biomarkers
Bayesian adaptive	Can formally incorporate prior information and reduce total sample size	Requires careful prior specification and early regulatory alignment	Programmes with meaningful prior data and strong statistical support
N‐of‐1 crossover	Patient serves as own control	Needs interpretable washout and outcomes that change on a practical timescale	Symptomatic or reversible effects in chronic conditions
Platform/master protocol	Shared infrastructure and comparator framework across multiple interventions	High governance and coordination burden	Multiple programmes in a shared disease area or pathway

Endpoint selection is particularly important in small rare‐disease trials. In primary hyperoxaluria Type 1, 24‐h urinary oxalate was used as the primary efficacy endpoint in ILLUMINATE‐A, because oxalate overproduction lies directly on the causal disease pathway and higher urinary oxalate is associated with renal outcomes [7]. In SOD1‐ALS, reduction in plasma neurofilament light chain supported accelerated approval of *tofersen* because it reflects neuroaxonal injury and was considered reasonably likely to predict clinical benefit, with confirmatory clinical evidence still required [15]. These examples illustrate how endpoint acceptability depends on biological plausibility, evidence linking to clinical outcomes and the regulatory pathway used.

Single‐arm studies with historical or external controls remain common in ultra‐rare disease. Their interpretability is strongest when untreated progression is relatively predictable, outcome measures are standardised and expected treatment efficacies are large relative to natural‐history variability [4, 70]. These conditions are met unevenly across rare diseases.

Bayesian approaches can improve inference by integrating small trial datasets with external information such as natural‐history data or earlier studies. Their value lies in making assumptions explicit and quantifying uncertainty, not in removing the small‐sample problem. Where prior evidence is sparse or heterogeneous, their benefit is limited [71, 72].

The reversibility and dose adjustability of ASOs, siRNAs and mRNA therapeutics can also support adaptive evidence generation through dose escalation and intra‐patient modification [1, 73]. However, this flexibility remains constrained by chemistry, delivery system, tissue exposure, biodistribution and prior class experience [73, 74].

N‐of‐1 and N‐of‐few strategies may be useful when within‐patient change can be measured over a suitable timeframe but are less applicable when progression is slow or washout effects are incomplete [75].

Platform and master‐protocol approaches offer a more scalable alternative by sharing infrastructure, comparators and evidentiary frameworks across related interventions [76]. These approaches are especially relevant when multiple therapies target a common pathway, mutation class or delivery platform, although their operational complexity remains substantial.

## 5 Regulatory Frameworks for Programmable Therapeutics

### 5.1 Regulatory Mismatch in Programmable Therapeutics

Programmable therapeutics expose a growing mismatch between NAT development and existing regulatory systems. Because evidence generation is structurally constrained in rare‐disease NATs, regulatory systems increasingly need to assess platform‐level predictability rather than treating each programme as entirely de novo. Current frameworks were largely developed for conventional small molecules and biologics, where each product is treated as a distinct pharmacological entity requiring largely independent evaluation. By contrast, many NATs are generated through sequence adaptation within partially conserved chemical, manufacturing and delivery platforms [77, 78].

ASOs, siRNAs, mRNA therapeutics and programmable editing systems differ in persistence, reversibility, tissue exposure and long‐term risk. However, within individual modality classes, properties such as pharmacokinetics, biodistribution, immunostimulatory profile and aspects of toxicology can become increasingly reproducible when chemistry, delivery route, dose range and tissue context remain stable [73, 74]. Repeating extensive product‐specific evaluation can therefore create duplication when new programmes are built upon highly conserved therapeutic scaffolds [79, 80, 81].

GalNAc‐conjugated siRNA therapeutics illustrate this tension. Multiple programmes demonstrate reproducible hepatocyte targeting, pharmacokinetic behaviour and dosing characteristics across different sequences [21, 82]. Yet foundational toxicology and pharmacology studies may still need to be repeated for each new candidate despite limited incremental mechanistic insight. Similar principles are increasingly emerging for GalNAc‐conjugated ASOs and other chemically stabilised oligonucleotide platforms.

These limitations become particularly important in ultra‐rare disease, where fixed evidentiary requirements may exceed the practical feasibility of developing therapy for very small patient populations. In inherited metabolic disorders, strong monogenic causality may support a clear mechanistic rationale, while small cohorts, incomplete natural‐history data and uncertain biomarker–outcome relationships still constrain clinical development [83]. Programmable therapeutics therefore challenge the assumption that every sequence‐modified therapy should require a fully independent evidentiary package, particularly when platform characteristics are well established [84].

### 5.2 Individualised Therapies and the Limits of Product‐Based Regulation

The limitations of conventional regulations are especially apparent in individualised or ‘*n* = 1’ ASO therapies. These therapies are designed for a single patient or small mutation‐defined subgroup, representing the most direct application of programmable precision medicine [85, 86, 87].

Milasen for Batten disease demonstrated that patient‐specific ASO can be designed, manufactured and administered within clinically meaningful timelines by building on established ASO chemistry and intrathecal delivery platforms [87]. Similar approaches are being explored in other ultra‐rare conditions [88]. Atipeksen, developed for a child with ataxia‐telangiectasia caused by a splice‐altering ATM variant, further illustrated how mutation‐specific ASOs can restore disease‐relevant splicing and support broader mutation‐targeted development [89].

These programmes highlight the regulatory tension. Although the therapeutic sequence is novel, platform features such as backbone chemistry, manufacturing, route of administration and aspects of toxicology may already be well characterised. Nevertheless, individualised therapies generally proceed through conventional investigational pathways requiring product‐specific toxicology and extensive documentation [88, 90].

For ultra‐rare diseases, the evidentiary burden required for a conventional development pathway may exceed the realistic scale, timeline or commercial viability of therapies intended for one or very few patients. At the same time, reducing evidentiary standards without an alternative framework risks undermining safety, reproducibility and public trust. This tension highlights why programmable therapeutics cannot be evaluated solely through traditional product‐based logic. Instead, the central regulatory question increasingly becomes which aspects of a therapeutic platform can reasonably be generalised and which risks remain sequence‐specific, target‐specific or disease‐specific.

Existing mechanisms such as compassionate use and expanded‐access programmes demonstrate that regulatory flexibility is possible [91]. However, these pathways currently function primarily as exceptional accommodations rather than components of a systematic framework for programmable therapeutics. Recent global experience further highlights the emerging transition from isolated N‐of‐1 interventions towards health‐system frameworks capable of supporting the equitable, sustainable and scalable development and delivery of individualised therapies [92].

### 5.3 Towards a Platform‐Informed Regulatory Architecture

A more adaptable regulatory approach would shift part of the evidentiary focus from individual products towards partially validated therapeutic platforms. Shared characteristics such as chemistry, manufacturing, delivery, pharmacokinetics, biodistribution and class‐related toxicology could support multiple related programmes [93, 94]. This would not reduce scrutiny, but allow regulators to distinguish established platform properties from novel biological risks such as sequence‐dependent off‐target effects, target biology, immunogenicity, durability and the relationship between molecular correction and clinical outcome.

This approach is most applicable where platform reproducibility is already established. Liver‐directed GalNAc‐siRNA or ASO therapeutics provide the clearest current example with sequence variation occurring within conserved chemical and delivery architectures associated with predictable hepatocyte uptake and pharmacological behaviour [73, 93]. Similar principles may progressively extend to other modalities as clinical experience accumulates.

One potential implementation strategy is the development of Platform Master Files (PMFs), containing validated information regarding platform pharmacology, biodistribution, manufacturing and toxicology. Individual submissions could reference this shared infrastructure while providing focused bridging data addressing sequence‐specific and disease‐specific considerations.

New sequence variants would be supported by focused bridging packages, including in vitro and in vivo evidence of target engagement, as well as sequence‐specific safety considerations. In parallel, real‐world evidence and post‐market data could be incorporated into the PMF, enabling continuous refinement of safety and performance understanding [48, 95].

This approach also aligns with emerging mechanism‐informed regulations. The U.S. Food and Drug Administration (FDA)'s Plausible Mechanism Pathway reflects growing interest in linking molecular mechanism, pharmacodynamic activity and clinically meaningful surrogate endpoints where conventional trials remain difficult [95, 96]. Programmable therapeutics fit this approach because their effects can often be traced from sequence‐specific interactions to molecular correction and measurable biological effects. Platform‐based evidence therefore offers a way to reduce assessment of established properties while retaining scrutiny of genuinely novel risks.

### 5.4 Ethical, Operational and Societal Considerations

Platform‐informed regulatory systems also raised ethical, operational and societal considerations. It may improve the feasibility of ultra‐rare disease and individualised therapies that are difficult to develop within conventional commercial and regulatory models. However, greater regulatory flexibility requires stronger long‐term monitoring, particularly for genome‐editing and other durable therapeutic modalities associated with delayed adverse events, irreversible effects and long‐term safety uncertainty [74, 97]. Consequently, platform‐informed regulation would require robust post‐market surveillance systems, shared longitudinal registries and coordinated real‐world evidence generation.

Clinical setbacks also provide important lessons for programmable therapeutics. In the SB‐913 MPS II programme, detectable hepatic genome editing did not translate into sustained IDS enzyme activity sufficient for clinical benefit, illustrating that molecular proof of mechanism does not necessarily establish therapeutic efficacy [98]. The 2018 human CCR5 embryo‐editing case further highlighted the ethical and governance risks associated with irreversible and heritable genome modification [99]. Together, these examples reinforce the need for rigorous benefit–risk assessment, transparent reporting, independent oversight and long‐term follow‐up for durable editing technologies.

Implementation will also require new forms of regulatory expertise and governance. Regulators will increasingly need to evaluate not only individual products but also evolving therapeutic platforms whose risk profiles change over time as additional clinical data accumulate. Shared evidence infrastructures, interoperable natural‐history datasets and international coordination may therefore become increasingly important components of future programmable medicine ecosystems [48, 95, 100].

## 6 Emerging Directions and Next‐Generation Platforms

NATs are transitioning from early proof‐of‐concept programmes towards a broader and increasingly scalable therapeutic paradigm. Emerging directions include expansion of NATs into broader disease areas and common chronic disorders, advances in extrahepatic delivery technologies, integration of computational oligonucleotide design and the gradual clinical emergence of programmable editing systems.

### 6.1 Expansion of Late‐Phase Clinical Development

Recent late‐stage clinical programmes demonstrate that NATs are progressing into neurological, cardiovascular and metabolic disorders, reflecting increasing confidence in their adaptability and reproducibility.

In Dravet syndrome, the Phase III trial of *zorevunersen* (developed by Stoke Therapeutics), an ASO to induce exon‐skipping of a poison exon in the *SCN1A* gene to produce more Nav1.1 sodium channel proteins, showed a substantial reduction in seizure frequency together with improvements in cognitive functions, behaviour and overall quality of life [101].

In Angelman syndrome, multiple Phase III programmes, including *ION582* (developed by Ionis Pharmaceuticals) and *GTX‐102* (developed by Ultragenyx/Gene Tx), use ASOs to target the antisense transcript that suppresses *UBE3A* expression [102, 103, 104]. The presence of parallel late‐stage clinical trials within a single disorder also suggests increasing maturity of ASO strategies across related paediatric neurodevelopmental mechanisms.

NATs are also increasingly entering large‐scale chronic disease indications, particularly in cardiovascular medicine. Although these programmes are not themselves rare‐disease therapies, they substantially expand long‐term safety experience, manufacturing scalability and regulatory familiarity. *Pelacarsen*, an ASO targeting lipoprotein(a) and *olpasiran*, a GalNAc‐conjugated siRNA directed against the same pathway, are currently undergoing cardiovascular outcomes trials designed to determine whether sustained biomarker reduction translates into decreased risk of major adverse cardiovascular events [105, 106]. These studies reflect an important shift towards establishing clinically meaningful outcome benefit at population scale.

### 6.2 Emerging Delivery Technologies Beyond the Liver

Despite substantial progress in liver‐directed delivery, efficient extrahepatic targeting remains one of the major limitations preventing broader NAT implementation. Current research is therefore increasingly focused on delivery systems capable of accessing skeletal muscle, heart, lung and central nervous system tissues.

Ligand‐conjugated oligonucleotide platforms are among the most promising approaches. Peptide‐, lipid‐ and antibody‐conjugated ASOs are being developed to enhance receptor‐mediated uptake into tissues that have historically been difficult to target systemically [107, 108]. Transferrin receptor 1 (TfR1) has emerged as a particularly attractive delivery target because it is expressed in skeletal muscle, cardiac muscle and brain endothelial cells and undergoes efficient receptor‐mediated internalisation or transcytosis [109]. Preclinical studies have demonstrated that TfR1‐targeted peptide and antibody conjugates can substantially improve oligonucleotide uptake and potency in muscle and CNS tissues [110].

Early clinical translation is now beginning to emerge. *Delpacibart etedesiran* (AOC 1001), a TfR1‐targeted antibody–oligonucleotide conjugate developed for myotonic dystrophy Type 1, has provided early evidence that systemic antibody‐mediated delivery can achieve skeletal muscle target engagement in humans [111]. Although these approaches remain early in development, they suggest that future NAT scalability may depend as much on delivery innovation as on advances in oligonucleotide chemistry itself.

### 6.3 Artificial Intelligence and Computational Oligonucleotide Design

Increasing programmability also creates opportunities for computational optimisation of NAT design. Machine learning and artificial intelligence (AI)‐based approaches are increasingly being used to improve sequence selection, predict off‐target interactions, optimise splice‐switching efficiency and model RNA secondary structure [112, 113].

These approaches may become particularly important for individualised and mutation‐targeted therapies, where rapid design iteration and prioritisation are essential. Computational modelling may also improve prediction of tissue‐specific activity, immunostimulatory potential and sequence‐dependent toxicology, thereby helping to reduce development timelines and experimental burden [114]. ASOptimizer, OligoAI and eSkip‐Finder are examples of newer oligo‐predicting AI models that are trained using publicly available data [114, 115, 116].

While these models are advancing in the right direction, AI‐assisted design does not eliminate the need for experimental validation. Biological activity remains strongly influenced by tissue context, delivery system, RNA accessibility and target biology. Nevertheless, computational approaches may progressively increase the scalability and reproducibility of programmable therapeutic development, particularly when integrated with evolving platform‐based regulatory frameworks.

### 6.4 Towards Programmable Therapeutic Ecosystems

The convergence of programmable oligonucleotide chemistry, increasingly reproducible delivery systems, computational design tools and platform‐informed regulatory thinking is gradually transforming NATs from isolated therapeutic products into broader programmable therapeutic ecosystems.

New interventions may increasingly build on validated chemistry and delivery frameworks rather than entirely de novo drug development. AI‐assisted sequence optimisation, expanding platform toxicology datasets, shared manufacturing infrastructure and growing regulatory familiarity may collectively accelerate the development of both population‐scale and individualised therapies.

Inherited metabolic disease may be particularly well suited to this transition because most disorders are monogenic, mechanistically well defined and associated with measurable biochemical biomarkers that can support mechanism‐informed development. The relative maturity of liver‐directed delivery platforms further strengthens the translational feasibility of NATs in metabolic disease compared with many extrahepatic conditions [2].

At the same time, programmability should not be mistaken for uniform maturity. Clinical predictability remains highly variable across tissues, modalities and disease contexts, particularly for extrahepatic delivery and durable genome‐editing approaches. Future progress will therefore depend not only on continued technological innovation, but also on stronger evidence infrastructure, long‐term safety monitoring and regulatory systems capable of distinguishing reproducible platform properties from genuinely novel biological risk [117].

## 7 Conclusion

NATs have rapidly evolved from experimental molecular tools into an increasingly mature therapeutic class capable of targeting a broad range of inherited metabolic and genetic disorders. Advances in oligonucleotide chemistry, delivery systems and molecular design have enabled clinically meaningful modulation of gene expression through ASOs, siRNAs, mRNA therapeutics and emerging programmable editing technologies.

As highlighted throughout this review, successful implementation of NATs depends not only on molecular programmability, but also on achieving appropriate therapeutic fit between disease biology, tissue accessibility, durability requirements and long‐term clinical context. Delivery feasibility, biomarker validation, natural‐history infrastructure and scalable evidence generation remain major determinants of translational success, particularly in ultra‐rare disorders where conventional development pathways are often difficult to apply.

At the same time, increasing platform reproducibility is beginning to challenge traditional product‐based regulatory paradigms. Because evidence generation is inherently constrained in rare‐disease NAT development, future regulatory systems will increasingly need to evaluate platform‐level predictability alongside product‐specific risk, rather than treating every programmable therapeutic as entirely de novo.

Emerging advances in extrahepatic delivery, computational oligonucleotide design, artificial intelligence‐assisted optimisation and programmable editing technologies further suggest that NATs are transitioning from isolated precision medicines towards broader programmable therapeutic platforms. Despite remaining challenges in delivery, long‐term safety, scalability and equitable access, NATs increasingly represent a scalable and mechanism‐directed approach to precision medicine for inherited metabolic and genetic disease.

## Author Contributions

T.T., S.S., P.G. and H.Z. were involved in drafting and proof‐reading this article.

## Funding

The authors confirm independence from the sponsors; the content of the article has not been influenced by the sponsors. The authors would like to thank funding support from the UKRI/MRC (MR/Y008405/1), the UKRI/Engineering and Physical Sciences Research Council (EP/Z536350/1), the LifeArc, The Royal Society (IEC\NSFC\211238), Rosetrees Trust (PGL24/100137) to H.Z.; the Great Ormond Street Hospital Charity (V4523) and the University College London Technology Fund (89‐315) to H.Z. and P.G.; the NIHR Great Ormond Street Hospital BRC award (23GI35)—Clinical Training PhD Fellowship Award to S.S, Oxford‐Harrington Rare Disease Centre and the Muscular Dystrophy UK, 22GRO‐PG36‐0552. All authors are supported by the NIHR Great Ormond Street Hospital Biomedical Research Centre. The views expressed are those of the author(s) and not necessarily those of the NHS, the NIHR or the Department of Health.

## Ethics Statement

The authors have nothing to report.

## Consent

The authors have nothing to report.

## Conflicts of Interest

The authors declare no conflicts of interest.

## Data Availability

Data sharing not applicable to this article as no datasets were generated or analysed during the current study.
